# Cosmetics and Cosmeceutical Applications of Chitin, Chitosan and Their Derivatives

**DOI:** 10.3390/polym10020213

**Published:** 2018-02-22

**Authors:** Inmaculada Aranaz, Niuris Acosta, Concepción Civera, Begoña Elorza, Javier Mingo, Carolina Castro, María de los Llanos Gandía, Angeles Heras Caballero

**Affiliations:** 1 Department of Chemistry in Pharmaceutical Sciences, Pharmacy Faculty, Complutense University, Plaza de Ramón y Cajal, s/n, 28040 Madrid, Spain; facosta@ucm.es (N.A.); mccivera@ucm.es (C.C.); belorza@ucm.es (B.E.); javier.mingo@ucm.es (J.M.); caroca03@ucm.es (C.C.); gandia.mllanos@gmail.com (M.d.l.G.); 2 Bifunctional Studies Institute, Complutense University, Paseo Juan XXIII, 1, 28040 Madrid, Spain

**Keywords:** chitin, chitosan, chitosan derivative, chitin derivative, oral care, skin care, hear care, marine resources, over-the counter-drug, polymer carrier

## Abstract

Marine resources are well recognized for their biologically active substances with great potential applications in the cosmeceutical industry. Among the different compounds with a marine origin, chitin and its deacetylated derivative—chitosan—are of great interest to the cosmeceutical industry due to their unique biological and technological properties. In this review, we explore the different functional roles of chitosan as a skin care and hair care ingredient, as an oral hygiene agent and as a carrier for active compounds, among others. The importance of the physico-chemical properties of the polymer in its use in cosmetics are particularly highlighted. Moreover, we analyse the market perspectives of this polymer and the presence in the market of chitosan-based products.

## 1. Introduction

A cosmetic is defined as any substance or preparation intended to be placed in contact with the various external parts of the human body (epidermis, hair system, nails, lips and external genital organs) or with the teeth and the mucous membranes of the oral cavity with a view exclusively or mainly to cleaning them, perfuming them, changing their appearance and/or correcting body odours and/or protecting them or keeping them in good condition [1]. That means that cosmetics are intended to be applied outside the body and no treatment against any specific disease can be claimed. These types of products are strictly regulated by different governmental agencies.

The term cosmeceutical was first coined by R.E Reed in 1962 [2]—Reed´s definition included four statements related to the idea of an increase in the quality of cosmetic products, which are included in the cosmetic industry nowadays (Table 1).

Nowadays, the use of the term cosmeceutical is widespread [3,4,5,6] with a different meaning that was established by Klingman in 1993 [7]. Cosmeceuticals are intended to carry out their functions as protection, whitening, tanning, anti-wrinkling, deodorants, antiaging and nail and hair care as a cosmetic product but cosmeceuticals claim to have biologically active ingredients with medicinal or drug-like benefits. In this new vision, the term cosmeceutical suggests that drugs and some cosmetics could share some possible borders which generates some controversial [8]. Cosmeceuticals include not only high-quality cosmetics as defined by Reed but also some prescription drugs such as topical retinoids, topical minoxidil, eflornithine and over-the-counter-drug (OTC) included in sun creams and antiperspirants.

Although it is claimed that a new regulation is needed to differentiate cosmeceuticals from cosmetics or drugs, these products have not been legally recognized yet. In fact, a consensus definition does not exit.

Since the antique, natural resources are well known as a source of biologically active substance to be used as cosmetics or cosmeceutical products. In recent years, much attention has been paid to marine resources as a new source of inexpensive and safe substances. Moreover, environmental issues are one of the main driven factors in the growing of this type of ingredients.

Form the active ingredients shown in Table 2, this review is going to focus on chitin, chitosan and their derivatives. Chitin is one of the most abundant polysaccharides found in nature which is widely distributed in the animal and vegetal kingdom. It appears in the exoskeleton of invertebrates such as crustaceans, mollusc or insects. Chemically chitin is a copolymer of *N*-acetylglucosamide and glucosamide. Chitosan is the *N*-deacetylated derivative of chitin which is only found in some fungi in nature. Chitosan is industrially produced from chemical *N*-deacetylation of chitin isolated from crustaceans. The main difference between chitin and chitosan is its solubility. Chitin is insoluble in aqueous media while chitosan is soluble in acidic aqueous systems being this property the criteria to differentiate chitin from chitosan [11]. Chitin and chitosan can be chemically modified using simple chemical tools producing a large number of derivatives with different properties that will be described in this review.

## 2. Target Organs for Cosmetics Products

**Figure 1:**
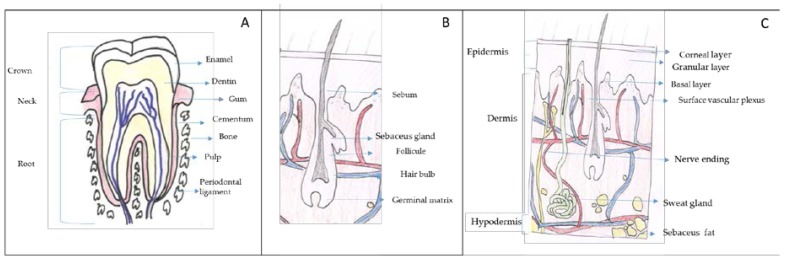
Target organs for cosmetic and cosmeceutical products. (**A**) Gums and tooth, (**B**) hair and (**C**) skin. Adapted from Wikipedia Commons (authors: KDS4444, Human tooth diagram-en.svg CC-BY-SA 4.0, Madhero88, Skinlayers.svg CC-BY-SA 3.0).

Chitin and chitosan can be used in different body sites such as skin, hair, gums and teeth. In this section, a brief anatomical description and the main problems/illness related to these body sites are described to better understand how chitin and chitosan can be used. In Figure 1, a brief description of these body structures is shown.

### 2.1. Gums

The periodontium is made up of four parts, namely tooth sockets (dental alveoli), periodontal ligament, cementum and gums (gingivae).

Gums, a soft connective tissue covered with mucous membrane, are found in the oral cavity that surrounds the necks of the teeth and adjacent alveolar bone (Figure 1A). The teeth are connected with the walls of the tooth sockets and anchored in the jaws by the periodontal ligament and the cementum. Each tooth consists of a hard shell that surrounds a cavity of soft tissue, known as pulp. The crown (the exposed part of the shell) is coated with a tough layer of enamel, beneath which is a layer of a yellowish substance similar to ivory, called dentin. The dentin and pulp form long, pointed roots that extend into the jawbone.

The gum tissue inflammation, known as gingivitis, causes the gum slightly detaches from the neck of the tooth. The space created between the tooth and the gum can then deepen to form a pocket where bacteria can rapidly build up. Gingivitis can be visible as bleeding gums but often does not cause any symptoms. Gingivitis can lead to periodontitis, an inflammation of the periodontium due to specific microorganisms that attacks the gums as well as the bone of the jaw. The presence of any of the 6–12 bacteria species responsible for the majority of the bacterial periodontitis can start periodontitis [12]. Advanced periodontitis can cause teeth to become loose and fall out or require them to be removed. It is very remarkable that illnesses related to the oral cavity are not only an aesthetic problem but also a serious health problem. The scientific evidence has shown a direct link between periodontal disease and cardiovascular disease [13]. The relationships between periodontal disease and other non-communicable diseases (hypertension, diabetes mellitus, osteoporosis, cerebral infarction, angina pectoris, myocardial infarction and obesity) have also been evaluated. This study demonstrated that the presence of periodontal disease is associated with a significantly elevated risk of non-communicable diseases in the Korean adult population, especially obesity, osteoporosis and angina pectoris [14].

### 2.2. Teeth

Dental caries and tooth wear are the main factors related to mineral lost and enamel demineralization. Dental caries is defined as a localized chemical dissolution of the tooth surface caused by metabolic events that occur in the dental plaque (biofilm that grows on surfaces within the mouth) [15]. The role of *Streptococcus mutans* is well recognized in the initiation of dental caries being attributed at least, in part, to its ability to colonise the tooth surface. Therefore, factors which prevent oral bacterial attachment to tooth are of considerable interest for the prophylaxis of this infectious disease.

Tooth wear is every mineral lost non-related to dental caries or dental trauma. Tooth wear can be produced by physical tooth-to-tooth contact (attrition), other physical contacts non-related to teeth (erosion) or chemical dissolution of tooth substance caused by acids, unrelated to the acid produced by bacteria in dental plaque [16]. Dental abrasion is a particularly worrying process in primary teeth, because the enamel layer in primary teeth is quite thin and, therefore, abrasions are frequently observed. With the age, the permanent teeth also accumulate dental abrasion. Dental erosion produces bulk mineral loss with a partly demineralized surface of reduced micro-hardness. Moreover, in early stages, coronal dentin often is exposed. The primary objective goal of active ingredients against dental erosion is to increase the resistance of tooth surfaces or pellicles to acids.

Several approaches including physical dental plaque remonition, use of antimicrobials and the use of fluorides are used to manage dental care. Caries-prophylactic agents may be delivered to the oral cavity by various delivery formulations (vehicles) such as mouth rinses, sprays, dentifrices, gels, chewing guns, lozenges and sustained-release formulations or devices (including nanoparticles/microparticles and functionalized films) which different characteristics are described in Table 3.

### 2.3. Hair

Hair is mainly composed of a protein called keratin. The second most abundant component are lipids, mainly ceramides. These lipids avoid the interfibre friction which is related to the sensory perception of hair. Hair shaft structure is very complex and consists of several concentric layers known as medulla, cortex and cuticle (Figure 1B). The medulla is the innermost layer, it is soft and fragile. Its function is unclear not appearing in some types of hair. The cortex is formed of elongated cells aligned along the axis of the fibre and it is filled with keratins that are arranged in a coiled-coil configuration. The cortex is responsible for the mechanical strength of hair and it controls water uptake. Moreover, the cortex contains melanin which accounts for hair colour.

Finally, the cuticle, the outer covering layer, is a thin laminar-like structure composed of layers of overlapping, flat, scale-like cells acting as a protective hair sheath. The cuticle is responsible for hair hydrophobicity since it is covered with a single molecular layer of lipids that makes the hair repels water.

Several factors account for hair protein denaturalization such as UV-exposition, heat stress from curling irons and blow dryers, chlorine, harsh chemicals in colouring, straightening and perming. Hair care products need to fulfil several requisites as summarized in Table 4.

### 2.4. Skin

The human´s largest body organ is the skin. The surface area of adults is about 1.6 m^2^ (female) to 1.8 m^2^ (male) [17]. The skin is composed of three layers: epidermis, dermis and hypodermis (Figure 1C) [18]. In the epidermis, there are five sublayers called *stratum corneum*, *stratum lucidum stratum granulosum*, *stratum spinosum and stratum germinativum* or *stratum basale*. The *stratum basale*, the deepest layer, is primarily made up of basal keratinocytes. These cells divide to form the keratinocytes of the *stratum spinosum*, which migrate superficially in a journey that takes approximately fourteen days and become flat, keratenised, water resistant dead cells of the corneum. At the transition between the *stratum granulosum* and the *stratum corneum*, cells secrete lamellar bodies (containing lipids and proteins) into the extracellular space. This results in the formation of the hydrophobic lipid envelope responsible for the skin's barrier properties. The *stratum corneum* forms a barrier to protect underlying tissue from infection, dehydration, chemicals and mechanical stress. Desquamation, the process of cell shedding from the surface of the *stratum corneum*, balances proliferating keratinocytes that form in the *stratum basale*.

The dermis is the structure beneath the epidermis, it is approximately 15 to 40 times as thick as the epidermis and it is composed of three layers (papillary layer, subpapillary layer and reticular layer). The components of the dermis comprise the fibrous tissue and the dermal matrix formed by cells in the interstitial components. Interstitial components are a dense network of structural proteins. Collagen fibres account for 70% of the weight of dry dermis; elastic fibres are composed of elastin. In between fibres and between cells a gelatinous amorphous substance called ground substance is observed. This matrix is mainly composed of proteoglycans and glycoproteins. The dermis is responsible for the skin´s elasticity and the senses of touch and heat.

The hypodermis or subcutaneous tissue is the layer between the dermis and the fascia and consist of a fat-rich tissue. The fat tissue acts to preserve neutral fat, cushion against external physical pressure, retain moisture and generate heat.

Three functions have been described for the skin: protection, regulation and sensation. The skin offers a protective barrier against harmful environmental factors, such as heat, solar ultraviolet (UV) irradiation, infection, injury and water loss. However, lifestyle and environmental factors cause both cosmetics and dermatological problems.

There are two primary skin ageing processes, intrinsic and extrinsic. Intrinsic skin ageing depends on genetic background, therefore seems to be inevitable and not subject to modification through changes in human behaviour. On the contrary, extrinsic ageing is caused by environmental factors such as light, heat, cold, etc. and therefore can be altered by modifying our style life.

**Figure 2:**
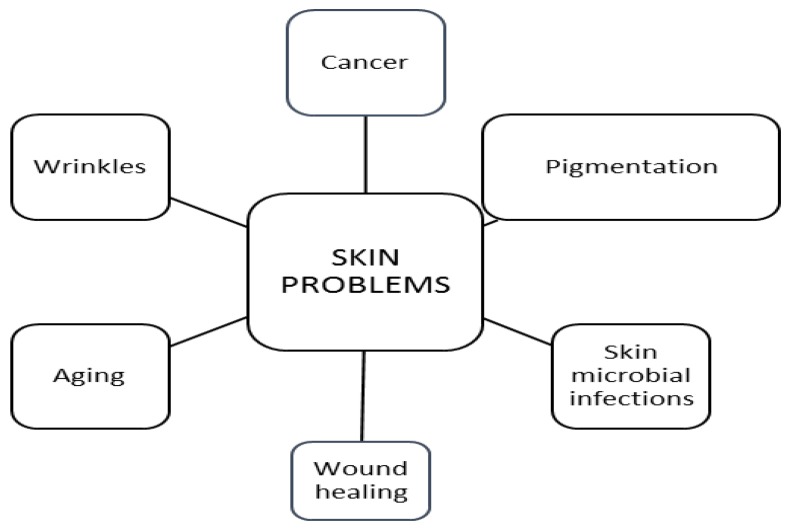
Major worldwide skin problems.

Skin cosmeceuticals have been developed after research on common skin problems like hyperpigmentation, skin cancer, skin microbial infections, wound healing and wrinkles associated with sun damage and ageing (Figure 2) [19].

## 3. Chitin, Chitosan and their Derivatives in Cosmetics and Cosmeceutical Industry

Chitin exhibits low solubility and it is quite difficult to handle. On the contrary, chitosan is soluble in acid aqueous solutions and can easily produce different conformations such as micro, nano and milli particles, films, scaffolds and fibres among others [20]. Oligosaccharides and some chitin and chitosan derivatives are water soluble at physiological pH and exhibit improved or even new properties. When taking into consideration the use of chitin, chitosan and derivatives in cosmetics one should keep in mind that these polymers can act as ingredients due to their specific properties or as a carrier of other active ingredients due to their technological properties.

The cosmetic ingredients are on the EU market regulated over Cosmetic Product Regulation adopted in 2009 [21]. CosIng is the European Commission database for information on cosmetic substances and ingredients (http://ec.europa.eu/growth/tools-databases /cosing/, last accessed 23 January 2018). A search using the term chitosan on this database retrieved 44 results while 8 results were retrieved with the term chitin.

According to CosIng database, the functions assigned to chitin are abrasive and bulking whereas the functions assigned to chitosan are film forming and hair fixing. A large number of functions, which will be described in this review, are identified for different chitosan derivatives. It is remarkable that not all activities and derivatives discussed in this review are included in CosIng database. Besides CosIng, this paper includes data from research journals and patents. Moreover, beyond the properties described in CosIng, other relevant biological properties of interest for the cosmeceutical can be found in chitin, chitosan and derivatives such as antifungic and wound healing activities, bio-adhesivity, non-toxicity and biodegradability [22].

### 3.1. Chitosan and Derivatives in Oral Healthcare

The use of chitosan has been proposed in all fields of dentistry including preventive dentistry, conservative dentistry, endodontics, surgery, periodontology, prosthodontics and orthodontics [23]. In this review, we are going to focus on the use of chitosan in preventive oral care. Oral healthcare is focused on preventive dentistry which aim is to avoid dental and gums illness.

**Figure 3:**
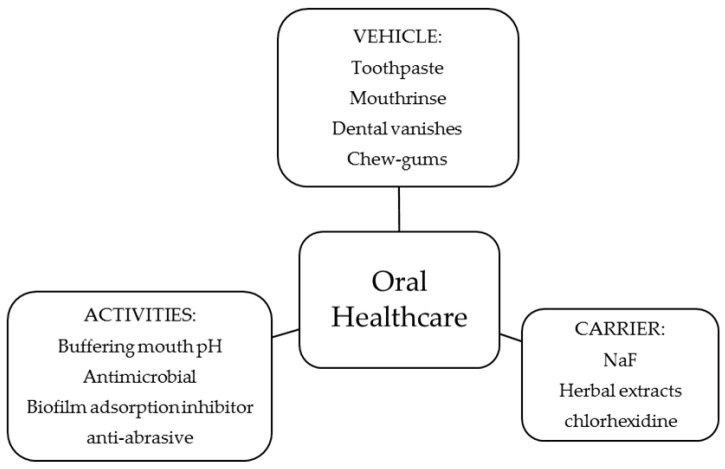
Use of chitosan and derivatives in preventive oral healthcare.

In Figure 3, a summary of the activity of the polymers in oral healthcare and the type of product tested is shown. In this section, a description of each activity and its relationship to the physicochemical properties of the polymers, when possible, is given.

#### 3.1.1. Reduction of Dental Plaque

Dental caries started via a carbohydrate fermentation process due to bacteria metabolism, in which the production of strong organic acids such as lactate, formate and pyruvate cause demineralization of the tooth surface. It has been reported that the optimum pKa value for buffering substances against plaque pH fall in vitro maybe around 6.3 [24]. This value falls in the pKa range of chitosan which is found around 5.1 and 6.5 depending on the chitosan *M*_w_ and degree of acetylation (DA). It has been described that pKa decreases with chitosan *M*_w_ while decreased with acetylation degree [25]. The effect of six low molecular weight chitosans (500–3000 Da) with acetylation degrees ranging from 0.05–0.5 were tested in vitro and in vivo for dental plaque pH reduction [26]. Evaluations using *S. mutans* cell suspensions in vitro revealed no differences among chitosan samples regarding their ability to reduce pH fall in dental plaque. Moreover, chitosan samples did not have any influence on the glycolytic activity of *S. mutans.* The clinical evaluation showed differences between the different chitosan samples. The most effective sample was a chitosan with a molecular weight of 3000 kDa and DA of 0.5. The second ranked had a *M*_w_ of 500 Da and DA of 0.05.

Another strategy to control dental plaque is the use of molecules with antimicrobial activity. The gold standard to avoid dental plaque and gingivitis is chlorhexidine gluconate (0.2%) due to its strong antimicrobial activity against *S. mutants*. However, this product has several side effects such as tooth staining, alteration in taste perception, changes in tongue sensitivity and pain due to the alcoholic content of the oral rinse. Moreover, chlorhexidine gluconate has a strong effect against *S. mutants* but little effect against other bacteria presented in dental plaque. Therefore, there is a great interest in new products with wide antimicrobial activity against oral pathogens with fewer side effects than chlorhexidine gluconate.

Chitosan has a wide antibacterial activity and therefore its activity against other bacterial strains, besides *S. mutants*, related to dental caries has been evaluated by several authors [27,28,29,30].

The evaluation of chitosan’s antimicrobial action mechanism testing two chitosan samples (624 kDa, DA < 0.25 and 107 kDa, DA 0.15–0.25) showed that both MWs acted upon the bacterial cell wall and were not capable of interacting with the intracellular substances, showing little to non eflocculation capability [29]. In Table 5, the reported minimal inhibitory concentration (MIC) values from different chitosan samples against different oral pathogen are summarized. In general, the higher the Mw of the polymers the lower was the MIC value. The exceptions were *P. buccae* and *P. intermedia*. Against *P. Buccae* the lower was chitosan´s *M*_w_ the lower was the MIC; while against *P. intermedia* the lowest MIC was observed with medium *M*_w_ chitosan samples.

The antimicrobial activity of chitosan (low *M*_w_, DA < 0.15) and chitosan nanoparticles *against S mutans*, *S sobrinus*, *S sanguis and S salivarius* was tested. The MIC of chitosan nanoparticles was lower in some strains than the MIC of chitosan [31]. This result is in good agreement with previous results that showed a better antimicrobial activity of chitosan nanoparticles when comparing to chitosan due to their small size [32]. The anti-inflammatory activity of chitosan nanoparticles was also studied [33]. This study showed that chitosan nanoparticles exerted a predominantly anti-inflammatory activity by modulating PGE2 levels through the c-Jun N-terminal kinases (JNK) pathway.

No antimicrobial activity was detected against *S. sanguis*, *S. gordonii*, *S. constellatus*, *S. anginosus*, *S. intermedius*, *S. oralis*, *S. salivarius and S. vestibularis* when low-molecular-weight produced by enzymatic chitosan depolymerisation was tested [34]. This result is contradictory to the previous one in which low Mw is claimed to exhibit antimicrobial activity against *S. sanguis*. In this case, authors did not describe properly the chitosan characteristics. Other authors have found that chitosan gels did not exhibit antimicrobial activity per se against *A. actinomycetemcomitans* and *S. mutans* but no data regarding chitosan properties were included in this paper [35].

Besides antimicrobial activity, other chitosan effects such as cell stimulation and biofilm adsorption inhibition have been reported. Chitosan (1080 kDa, DA 0.14) stimulated the proliferation of human periodontal ligament cells [27]. Chitosan micro and nanoparticles were well tolerated by gingival fibroblasts and were able to stimulate cell proliferation through the Ras-Erk-ETS 1/2 (ERK1/2) signalling pathway. Furthermore, it was hypothesized that a synergistic response between chitosan particles and growth factors (such as PDGF-BB) may stimulate cell proliferation in gingival fibroblasts [36].

The inhibition of *S. mutant’s* attachment to hydroxyapatite (HA) and saliva-treated hydroxyapatite (S-HA) by low *M*_w_ chitosan produced by enzymatic depolymerisation (membrane cut-off 1500) has been described. When S-HA and HA beads were treated with low molecular weight chitosan, a reduction in *S. mutans* adsorption ranging from 47 to 66% was observed [37]. The effects of chitosan Mw upon *S mutant*’s adherence and biofilm formation was studied testing two chitosan samples (High Mw sample: 624 kDa, DA < 0.25 and medium *M*_w_ sample 107 kDa, DA 0.15–0.25) [38]. Both samples showed an evident strong effect on *S. mutans* adherence and biofilm formation, inhibiting both adherence and biofilm formation in the initial stage of dental plaque formation as well as disrupting mature biofilms. The high Mw sample exhibited better performance regarding inhibiting dual-species biofilm formation. The effect of chitosan *M*_w_ (0.8–6 kDa) and DA (0.05–0.90) on the adsorption of *S. sobrinus* to S-HA was tested. The inhibition of *S. sobrinus* adsorption on S-HA showed a positive correlation with the Mw of chitosan and the optimal degree of acetylation was determined to be 0.4–0.5 [39].

Chitosan has been added to tooth pastes, rinses and other vehicles to test its activity against dental plaque formation in real formulations. Chitosan included in tooth paste and mouthwashes to avoid biofilm formation due to the presence of *S mutans* in the mouth demonstrated a reduction of *S mutans* colonies in early childhood caries [40]. Moreover, daily use of chitosan rinse (6 kDa, DA 0.4, 0.5% *w*/*w*) was effective to reduce dental plaque formation and count of salivary *S mutants* [41]. A chitosan mouthwash, composed of a mixture of two chitosans (DA < 0.25; *M*_w_ 624 kDa and DA 0.15–0.22; *M*_w_ 107 kDa, the final concentration of either chitosan being 0.4% *v*/*v*), was compared with two commercial mouth rinsers [42]. The chitosan-based product exhibited a wide range of antibacterial activity (against *S. mutans*, *E. faecium*, *P. intermedia* and *L. acidophilus*) and antifungal activity against *C. albanis.* Moreover, its activity was superior to both commercial mouthwashes examined. In a further study, chitosan mouthwash safety was evaluated and the antimicrobial activity was corroborated through in vivo assays [43]. Chlorhexidine gluconate showed minor efficacy on other cariogenic bacteria than *S. mutans* such as *S. sanguinis* or *lactobacilli* even when applied at high concentrations [44]. The effect of two chitosan samples, at a concentration 0.2% which corresponds to the chitosan MIC for oral *streptococci* according to previous studies, alone or in combination with chlorhexidine gluconate on dental plaque was studied. The mixtures containing chitosan and chlorhexidine exhibit a better antibacterial activity than chlorhexidine alone. Differences were observed regarding the chitosan sample used but unfortunately, the polymer characteristics were not described [45]. In another study, chlorhexidine gluconate mucoadhesive gels were produced with different chitosan samples (High *M*_w_ (1400 kDa, DA 0.2) and medium Mw (272 kDa, DA ranging from 0.05 to 0.27). In this case, the combined gels exhibited lower MIC than pure chitosan or chlorhexidine gluconate against *P. gingivalis*. Interestingly, the best combined formulation did not correspond to the chitosan that exerted more remarkable antimicrobial activity alone [28]. A chitosan tooth paste containing herbal extracts with antimicrobial activity against dental pathogens was formulated. The pharmaceutical evaluation of toothpaste was carried out as per the US Government Tooth Paste Specifications. After 4-weeks experiment the chitosan-containing polyherbal toothpaste significantly reduced the plaque index and bacterial count during in vivo tests [46]. Chewing gums containing chitosan demonstrated its ability to suppress bacterial growth and to increase salivary secretion *in vivo* which may improve the quality of life of dry-mouth patients. These findings suggest that a supplementation of chitosan to gum is an effective method for controlling the number of cariogenic bacteria in situations where it is difficult to brush one's teeth [47,48].

Besides chitin or chitosan, several derivatives of both polymers have been synthetized and tested against different bacterial strains. As seen in Table 6, most of these derivatives have antimicrobial activity against *S. mutants* while only some derivatives have exhibited activity against other *streptococci* strains [27,34,37,49,50,51,52].

#### 3.1.2. Reduction of Dental Abrasion

Another use of chitosan in dentistry is related to avoid dental abrasion. Dental tooth, which otherwise is beneficial to avoid dental plaque, contain abrasive components that may cause brush scratches or micro wear on sound enamel surfaces. It is thought that the presence of fluoride may cause less brushing abrasion on teeth with artificial decay lesions than toothpaste without fluoride since fluoride has a remineralization effect. Different ingredients, such as polyvalent cations (Sn^2+^, nano-phosphate salts (Ca/P), proteins and biopolymers such as chitosan, have been proposed to design more effective formulations as fluoride substitutes or included in fluoride tooth pastes [53].

A tooth paste containing chitosan (Chitodent^®^) was compared with a propolis-containing tooth paste (Aagaard^®^), a fluoridated (500 ppm) tooth paste (Elmex^®^) and a control group without treatment. The brushing abrasion depths formed in primary tooth enamel caused using the different tooth paste were compared. In this study, both propolis tooth paste and chitosan tooth paste showed an average brushing abrasion value on healthy surfaces lower than the observed using the fluoride tooth paste [54]. Divalent cations such as Sn^2+^ in combination with fluoride are more effective than fluoride alone to prevent dental erosion. The use of Sn^2+^ combined with chitosan and fluoride was proposed not only in terms of anti-erosive activity but also as anti-abrasive. The F/Sn/chitosan toothpaste (1.400 ppm F^−^, 3.500 ppm Sn ^2+^, 0.5% chitosan) reduced the erosive/abrasive tissue loss significantly compared to placebo (tooth paste without fluoride, Sn^2+^, chitosan) and showed an efficacy in the order of the positive control (GelKam = 3.000 ppm Sn^2+^,1.000ppm F^−^) or experimental toothpaste (1.400 ppm F^−^, 3.500 ppm Sn^2+^). The effect of chitosan was ascribed to its ability to bind to enamel creating a protective shell [55].

#### 3.1.3. Chitosan as Vehicle in Oral Healthcare

Chitosan has also been used as a vehicle for other therapeutic products with different activities. For instance, chitosan gels containing herbal extracts showed a dental plaque reduction of 70% and a reduction of bacterial counting of 85% [46]. Chitosan microparticles containing NaF produced by spray drying exhibited bioadhesive properties in the oral cavity acting as a fluoride reservoir both in fluoride burst release or fluoride controlled delivery systems [56]. Chitosan nanoparticles (100 nm) loading NaF were produced by ionotropic gelation using tripolyphosphate (TPP) as crosslinker agent [57]. Dental varnishes containing chitosan nanoparticles as NaF carrier was developed with antimicrobial activity against *S. mutants* and the ability to inhibit demineralization was proved [58]. Chitosan (DA 0.15–0.25 viscosity 200–800 cps) microparticles containing chlorhexidine diacetate were produced by spray-drying. Chlorhexidine-chitosan microspheres were dissolved faster in vitro than chlorhexidine powder. The antimicrobial activity was tested against *Escherichia coli*, *Pseudomonas aeruginosa*, *Staphylococcus aureus* and *C. albicans*. Buccal tablets were prepared by direct compression of the microparticles with mannitol alone or with sodium alginate. After their in vivo administration, the determination of chlorhexidine in saliva showed the capacity of these formulations to give a prolonged release of the drug in the buccal cavity [59].

Chitosan- hydroxypropyl methylcellulose 3D hydrogels containing O-toluidine for antimicrobial photodynamic inactivation were produced and tested against *S. aureus*, *A. actinomycetemcomitans* and *P. gingivalis* biofilms*.* These hydrogels showed promising results regarding their clinical use with an appropriate delivery of o-toluidine [60]

### 3.2. Chitin, Chitosan and Derivatives in Haircare

The usage of polymers in haircare products is gaining attention due to their ability to improve the rheological behaviour of the product or to enhance the adhesion of other ingredients to the hair. As previously mentioned in Section 2.3, the proteinic structure in damaged hair is denaturalized by different processes. It has been reported that the use of cationic polymers can help in the treatment of damaged hair. Polymers need to fulfil some requirements to be considered appropriate for hair care. As seen in Table 7, chitosan fulfils most of the requirements while each chitosan derivative needs to be addressed individually for most of them.

#### 3.2.1. Chitosan and Derivatives as Hair Care Ingredient

Chitosan and its derivatives have been included in a large variety of hair products such as shampoos, rinses, permanent wave agents, hair colorants, styling lotions, hair sprays and hair tonics [61].

Chitosan and its cationic derivatives have the ability to interact with keratin forming transparent, elastic films over hair fibres. These films increase hair softness, hair strength and avoid hair damage. Chitosan was blended with hyaluronic acid and collagen and the produced films on hair were studied. The covering of hair led to an increase in hair thickness and to the improvement hair mechanical properties with an enhancement in the general appearance and conditioning of the hair [62]. Beyond chitosan's filmogenic properties, chitosan gelling ability in hydroalcoholic mixtures was used to formulate chitosan in gel form [61,63]. Chitosan salts in different formulations such as hair lotions, hair conditioners and hair shampoos were tested as haircare products taking advance of the filming properties, emulsifier activity and cationic surface, respectively [64,65]. Chitosan, microcrystalline chitosan and quaternized chitosan were added to shampoos and hair sprays due to its film former activity and moisturizing effect [66,67,68]. Glycerol chitosan was included as a component into liquid hair strengthens or hair sprays due to their improved solubility and film forming capacity [69] and glyceryl chitosan forms foam and has an emulsifying action, so it could be used directly in shampoos [70].

Alkyl-hydroxypropyl-substituted chitosan derivatives were added as a component of hairsprays in substitution of synthetic resins in order to avoid long drying time, hair sticky feeling and helmet formation [71]. Moreover, the solubility in organic media allowed the use of halogen free propellant gases and their solubility in basic media allowed their use in alkaline permanent wave agents or hair colouring agents. Quaternary chitosan derivatives with solubility in aqueous and basic media were also developed for the same purpose [72,73].

Chitosan hair fixing, hair conditioning and hair filming functions, as well as Carboxymethyl chitosan gel forming functions, are described in CosIng database. Moreover, hair conditioning and film forming functions have also been described for a large number of chitosan derivatives (Table 8).

#### 3.2.2. Chitosan as a Vehicle in Hair Care

Androgenetic (or pattern) alopecia is a very common disease affecting both male and female. It is a genetically determined disorder characterized by the gradual conversion of terminal hairs into indeterminate and finally into vellus hairs. The standard treatment for male and female androgenic alopecia is Minoxidil sulphate in topic treatment. Minoxidil solubility in water is low and the formulations in the market included large amounts of ethanol or propylene glycol which are highly irritating in continuous use. Moreover, due to its potent antihypertensive activity dermal exposure and a consequent systemic effect should be avoided to minimize adverse side effects. Therefore, formulations with capacity to target minoxidil to hair follicles in a sustained manner are very desirable. Minoxidil sulphate was encapsulated in chitosan microparticles and nanoparticles. Chitosan microparticles were produced by spray-drying (Medium Mw chitosan 190–310 kDa, DA: 0.15–0.25). Even after swelling, the microparticles presented an appropriate diameter for drug target to hair follicle with a sustained release of the drug [74]. Minoxidil loaded chitosan nanoparticles (Low Mw chitosan, DA 0.15–0.25) provided a sustained release avoiding dermal exposure with a minoxidil two-fold increase in the follicle when compared to a controlled drug solution [75].

### 3.3. Chitin, Chitosan and Their Derivatives in Skin Care

**Figure 4:**
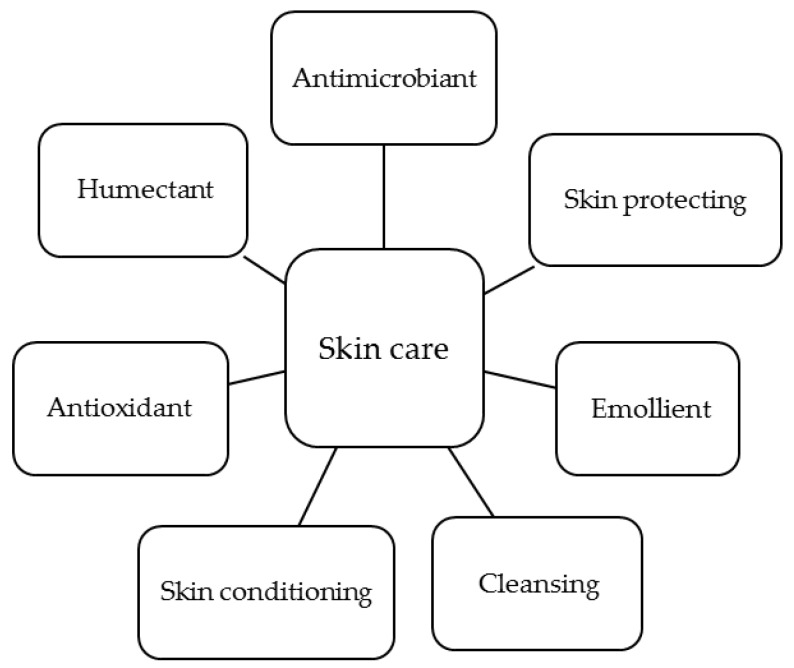
Main functions of chitosan derivatives in skin care.

The interest of the cosmetic and cosmeceutical industry on chitin, chitosan and their derivatives rely on the unique biological and technological properties of these polymers. Their main functions in skin care are summarized in Figure 4. In this section, these functions are reviewed and a general overview of the use of these polymers as vehicles for active ingredients is given.

#### 3.3.1. Application of Chitin, Chitosan and its Derivatives in UV Protection

Skin photoaging is a premature skin-aging damage after repeated exposure to ultraviolet (UV) radiation, mainly characterized by oxidative stress and inflammatory disequilibrium, which makes skin show the typical symptoms of photoaging such as coarse wrinkling, dryness, irregular pigmentation and laxity. Moreover, UV exposition is directly related to skin cancer.

UV spectra of chitosan and chitosan films reveal absorption below 400 nm and therefore they may be used as sunscreens. A chitosan gel with an in vitro Sun Protector Factor (SPF of 0.89) was reported [76]. When comparing two chitosan films, the transmittance of mushroom chitosan film for UVA-UVB (300–250nm) was lower than that of shrimp chitosan film. This result implies the capacity of UV resistance of chitosan extract from mushroom was better than that of shrimp chitosan film. Whether this difference is due to chitosan origin or due to other properties (*M*_w_ or DA) is not possible to be determined since no proper polymer characterization was carried out [77].

Chitosan oligosaccharides demonstrated its ability to reduce skin photoaging in hairless mouse dorsal skin after UV radiation for 10 weeks. Results indicated that chitooligosaccharides were able to regulate antioxidant and anti-inflammatory status [78].

Although along this review little examples of the use of chitin in cosmetic has been shown, due to its poor solubility in most aqueous and organic solvents, several examples regarding the use of chitin as protective UV molecule can be found in the literature as nanofibers and nanocrystals.

The potential use of chitin nanofibrils (NF) and chitin nanocrystals (NC) as components of skin-protective formulations was evaluated. The application of nanofibrils and nanocrystals to skin improved the epithelial granular layer and increased granular density. Furthermore, NF and NC application reduced the production of transforming growth factor beta (TGF-β) compared to that of the control group [79].

It is well known that urocanic acid is a major ultraviolet (UV)-absorbing chromophore. Chitin nanofibers (NFs) were produced by acid hydrolysis with urocanic acid and the protective effect of these urocanic acid chitin NFs (UNFs) and acetic acid chitin NFs (ANFs) against UVB radiation was tested [80]. A lower UVB radiation-induced cutaneous erythema than in the control was observed and sunburn cells were rarely detected in the epidermis of UNFs-or ANFs coated mice after UVB irradiation. These results showed that ANFs also exhibited a protective effect against UVB. This could be explained considering the inherent anti-inflammatory and antioxidant activity of chitin nanofibers.

#### 3.3.2. Use as Skin Cleansing, Skin Conditioning and Emollient

Cleansing products are those to help to maintain the body surface clean. Chitosan argininamide is the only chitosan derivative identified as skin cleansing according to CosIng database. Skin conditioning products are those which function is to maintain the skin in good conditions. A large number of chitosan derivatives and some chitin derivatives have been classified as ingredients with skin conditioning function (Table 9). Emollients function is to soften and smooths the skin. Some chitosan derivatives present this function whereas no chitin derivative has been described as emollient.

#### 3.3.3. Use as Humectant and Moisturizing Agent

Humectants are cosmetics intended to increase water content on top layers of the skin. Cationic humectants absorb to the negatively changes skin surface. As a humectant, chitosan was combined with pyrrolidone carboxylic acid (PCA) producing a film-forming humectant material [81]. Acyl and alkylated chitosan derivatives were produced to improve chitosan humectant properties [82]. Other chitin and chitosan derivatives with humectant function are summarized in Table 10. Humectant polymers activity depends on the cationic charge, molecular weight and hydrophobicity of the polymer [83]. In the case of chitosan, therefore samples with low DA and Mw may exhibit better humectant properties.

Moisturizing products increase the water content of the skin and helps keep it soft and smooth. Several chitosan samples with different *M*_w_ (1.2 × 10^3^ to 30 × 10^4^ kDa) were prepared by oxidative degradation with H_2_O_2_. The moisture-absorption and moisture-retention capacities of resulting chitosans were dependent on both the molecular weight and the degree of acetylation (DA). The moisture-absorption capacity increased as the *M*_w_ decreased. As the *M*_w_ was reduced the moisture- retention capacity first increased and then declined with a maximum moisture-retention capacity when the chitosan sample of 0.45 × 10^4^ kDa was used. Moreover, the moisture retention and moisture-absorption increased with chitosan DD [84]. From all tested samples, the best sample was a chitosan of 0.45 × 10^4^ kDa and a DA of 0.1 

When a family of carboxymethyl derivatives were tested (6-O-CM-Chitin, 6-O-CM-Chitosan, 6-3-O-CM-Chitin, 6-3-O-CM-Chitosan and *N*-CM-chitosan all samples showed good moisture-retention ability and the moisture-absorption properties were quite similar to those of hyaluronic acid. Results indicated that substitution in 6-OH position is the main active site for moisture absorption and retention with a lower contribution of 3-OH and *N* position. In 6-O-CM-Chitin and 6-O-CM-Chitosan as higher was the molecular weight, the better moisture-retention ability was observed [85]. Because chitosan has lower cost, this polymer might compete with hyaluronic acid as moisturizing agent in cosmetics.

Besides chitosan derivatives, some chitin derivatives also have moisturizing and humectant properties (Table 10). Moreover, chitin–glucan complexes, that are composed of chitin and beta-glucan units covalently linked, exhibited appropriated properties regarding basic moisturization in creams and after prolonged used no erythema was observed. Furthermore, skin physiology was positively modified and as a consequence some signs of skin ageing were improved [86]. Quaternized carboxymethyl chitosan-montmorillonite nanocomposites also exhibited appropriate properties against skin ageing. Not only due to their moisturizing effect but also due to their good UV-protection ability [87].

#### 3.3.4. Use as Surfactant, Emulsifier, Stabilizer and Viscosifier

Surfactants are very useful in the cosmetics industry being their function to lower the surface tension of cosmetics as well as to aid the even distribution of the product when used.

Chitosan yields stable water-in-oil-in-water (w/o/w) multiple emulsions without adding any surfactant. Emulsification of sunflower oil by chitosan solutions with the degree of acetylation (DA) between 0.25 and 0.05 was studied. DA only affects droplet size distribution which was unimodal at high DA and at low DA, for all tested concentrations. On the contrary at intermediate DA, distribution was unimodal only when we used the most concentrated solutions. Chitosan DA did not affect emulsion stability or ageing [88].

Chitosan can interact with anionic surfactants to form complexes which exhibit interesting surface-active properties related to surface tension and viscoelastic properties, even at very low surfactant concentrations (much lower than the CMC of pure surfactant). These properties allow their use as emulsion stabilizers in cosmetic formulations [89]. Chitosan derivatives containing fatty acid chains such as Chitosan Lauramide Succinamide, Chitosan Lauroyl Glycinate, Hydrolysed Chitosan Ferulyl Linoleate have been described as stabilizers in the CosIng database (Table 11).

Viscosity controlling ingredients are useful in the cosmetic industry to increase or reduce the viscosity of formulations. Viscosity can be modified by controlling the polymer concentration or the polymer Mw. The effect of chitosan Mw on the viscosity of a vitamin E-containing cream was studied. The apparent viscosity of vitamin E-containing creams increased with increasing chitosan Mw. At 0.5% *w*/*w* chitosan concentration, the apparent viscosity was higher than the control (2% glycerol monostearate). Apart from higher viscosity, larger storage life and better skin hydration than the control was observed [90].

*N*-Carboxybutyl chitosans were soluble in water and water ethanol mixtures giving more viscous solutions than the corresponding chitosans [91].

#### 3.3.5. Use as Antioxidant and Antimicrobial Agent

Antioxidant activity of chitin, chitosan and their derivatives can be attributed to in vitro and in vivo free radical-scavenging activities [92]. This activity is very valuable from the point of view of skin protection against oxidative damage. Moreover, their use as additive may prevent the oxidation of other active ingredients such as essential oils or vitamins. Antioxidant activity is particularly remarkable in the case of chitooligosaccharides (COS) [93,94]. COS, enzymatically produced, antioxidant activity depended on the type of enzyme used to hydrolyse chitosan (Chitosanase or lysozyme). COS produced by chitosanase have better antioxidant activity compared to COS produced with lysozyme. This behaviour seems to be related to the different distribution of acetylglucosamine and glucosamine residues in the COS backbone due to the different cleavage site of each enzyme [95]. Chitosan has been widely modified with well-known antioxidant molecules such as ferulic acid, glycolic acid, ascorbic acid and salicylic acid to improve its antioxidant activity (Table 12).

Chitosan and some derivatives (mainly cationic derivatives) exhibit antimicrobial activity against gram positive and gram negative bacteria [96]. Chitosan (low *M*_w_, viscosity below 30 mPa·s. at pH 5 and degree of acetylation most preferable to be at least 0.10) has been added in a fine emulsion for use as preservative in home care or personal care products against microbial spoilage [97]. Moreover, due to antibiotic resistance and chitosan activity against *Propionibacterium acnes* and *Staphylococcus aureus*, this polymer was evaluated as possible antimicrobial molecule in acne vulgaris. To determine the effect of molecular weight on antibacterial activity, chitosan of low Mw (50–190 kDa), medium *M*_w_ (190–310 kDa) and high *M*_w_ (310–375 kDa) was tested against *P. acnes* and *S. aureus.* The sample with the highest *M*_w_ had greater effect against both strains, particularly against *S. aureus* [98].

#### 3.3.6. Chitosan and Derivatives as Vehicle for Active Ingredients in Skin Care

Chitosan is a good polymer matrix for the delivery of active ingredients. Taking advance of the extraordinary technological properties of this polymer, chitosan has been processed into different formulations such as gels, micro and nanoparticles etc.

Several examples of sunscreen molecules included in chitosan formulations can be found in the literature. Phenylbenzimidazole sulphonic acid (PBSA) is a relatively photostable UV-B filter that was successfully encapsulated into chitosan microparticles with an encapsulation yield higher than 70%. The UV screening effect of a chitosan gel was increased by incorporating the microparticles containing PBSA into the gel [76]. In another example, benzophenone-3 was loaded into polycaprolactone nanocapsules coated with chitosan and the release was tested. Penetration profiles showed that a higher amount of benzophenone-3 remained at the skin surface and a lower amount was found in the receptor compartment after the application of the formulation containing chitosan-coated nanocapsules compared to a formulation containing its free form [99]. An oil-in-water photoprotective and antioxidant nanoemulsion (NE) containing chitosan was developed with the aim of protecting skin against ultraviolet radiation while toxic sunscreens substances included in the formulation are retained in the skin. Several molecules were tested: benzophenone-3, diethylamino hydroxybenzoyl hexylbenzoate, octocrylene and octylmethoxycinnamate, pomegranate antioxidant extract and chitosan. Formulations containing chitosan were stable for at least 6 months, were photostable when irradiated in a solar simulator and effective. Moreover, chitosan promoted retention of the formulation in the epidermis, thus increasing formulation safety [100]. A multifunctional hydroxyapatite-chitosan gel that works as an antibacterial sunscreen agent for skin care was developed. This gel conferred protection against UV-radiation and antibacterial activity [101]. Chitin Nanofibril-Hyaluronan nanoparticles (CN-HA) has the ability of easily loading active ingredients, facilitating penetration through the skin layers and increasing their effectiveness and safety as an anti-aging agent. CN-HA nanoparticles were evaluated in vitro measuring their antioxidant capacity, anti-collagenase activity and metalloproteinase and pro-inflammatory release. The efficacy was also shown in vivo by a double-blind vehicle-controlled study for 60 days on 60 women affected by photo-aging [102]. Finally, mycosporines and mycosporine-like amino proteins were grafted to chitosan to produce multifunctional materials based only in natural components. These materials were biocompatible, photoresistant and thermoresistant and exhibited a highly efficient absorption of both UV-A and UV-B radiations [103].

Retinols have shown promising results for photoaging and depigmentation at high concentrations which are higher than those found in OTC products. For acne treatment, the evidence is more limited and more studies are needed [104]. Formulations of retinols into appropriate vehicles may improve control delivery, stability, bioavailability and potency. Several chitosan formulations have been proposed with this aim. Retinol-encapsulated chitosan nanoparticles (100 nm) were produced using a water soluble chitosan (18 kDa, DA 0.04), these nanoparticles protect retinol against degradation [105]. Retinol was encapsulated in zein nanoparticles (300 nm) that were coated with chitosan. Chitosan Mw affected the particle size and polydispersity which increased as the Mw increased. On the contrary, a slight reduction of encapsulation efficiency was observed when Mw increased. This system improves retinol photostability [106]. In another example, retinol was encapsulated into succinic-chitosan nanoparticles by means of retinol complexation with the chitosan derivatives through H-bonding. The antioxidant activity of the encapsulated retinol was significantly greater than pure retinol [107].

Chitin Nanofibrils/bio-lignin non-woven tissues were electrospun with different antiaging ingredients. The beauty masks were controlled for their safeness and effectiveness both in vitro on keratinocyte and fibroblast cultures and in vivo on 30 volunteer women showing signs of premature skin aging (photoaging) for a period of 30 days. Results showed that the beauty masks that were not only effective on the aged and sensitive skin but also very safe and stable for a long period of time because they were free of water [108].

Polyphenols with a strong antioxidant activity have also been encapsulated in nanoparticles and mircrospheres produced by ionic gelation and by spray-drying, respectively [109].

Chitosan is also a useful matrix to encapsulate essential oils. For instance, Mentha Piperita essential oil was encapsulated in chitosan beads Two chitosan samples (High Mw chitosan 2.000 kDa, DA < 0.25 and medium *M*_w_ chitosan 750 kDa, DA 0.15–0.25) were used as polymeric matrix to encapsulate the oil. Chitosan glycolic solutions (High Mw, Low Mw and mixture of both chitosans) containing the essential oil were dropped to NaOH or TPP solutions. NaOH beads showed much better swelling properties and greater release of the essential oil. Encapsulation efficiency was not affected by chitosan sample and during beads breakage assay, medium Mw chitosan-based particles showed better behaviour [110].

## 4. Functional Characterization of Chitin and Chitosan in Cosmetics

There is a large variety of chitin and chitosan samples differing in polymer molecular weight and its distribution, deacetylation degree, acetyl distribution and crystallinity among other physical-chemical properties. Moreover, a myriad of chitin and chitosan derivatives have been produced from different chitin and chitosan samples by taking advance of the simplicity of chemical modification of these polymers. The wide range of applications in different fields is sustained by this structural variability. The question that arises at this point is which sample is the most appropriate for a specific application. Therefore, the proper sample characterization is an essential issue that needs to be carefully considered. Ambiguities in research studies regarding different properties and functions of these polymers are frequently found in the literature, these ambiguities coming, at least in part, from inadequate data on the polymer characteristics. During the elaboration of this review, we have frequently found research work in which the polymer samples were not characterized or generic data were given, such as deacetylation degree and molecular weight in a range rather than a specific value. It is also worth mentioning that accurate determination of the polymer characteristics is difficult since most of the proposed techniques are easy and simple but not accurate [111].

Keeping in mind these considerations, in Table 13, some general recommendations are included to assist researchers and industrials to select the most appropriate polymer for a specific purpose.

It is worth mentioning that differences can be observed between in vitro and in vivo assays due to the more complex environment found in in vivo assays. For instance, no effect of chitosan properties in its mouth pH buffering activity was observed in vitro while in vivo a specific chitosan performance a better behaviour as described in Section 3.3.1.

It has been reported that antibacterial activity depends on several parameters such as molecular weight, the degree of acetylation, the type of substituents and the type of bacterium. The exact mechanism of the antimicrobial action of chitin, chitosan and their derivatives is still unknown. Both low and high *M*_w_ polymers exhibit antimicrobial activity but different mechanisms have been proposed for each family of chitosan samples. High molecular weight polymers seem to form a film that protects cells against nutrient transport through the microbial cell membrane. Low molecular weight chitosan derivatives are water soluble and can better incorporate the active molecule into the cell. Gram-negative bacteria have an anionic bacterial surface on which cationic chitosan derivatives may interact electrostatically. Gram-positive bacteria seem to be inhibited by the binding of lower molecular weight chitosan derivatives to DNA or RNA. Chitosan nanoparticles exhibit an increase in loading capacity and efficacy [112]. Antibacterial activity of chitosan against oral pathogens seems to be mainly affected by chitosan molecular weight with better activity as the *M*_w_ increase. Antimicrobial activity against skin pathogens showed a similar trend although the evidence is lower since little research regarding chitosan characteristics has been carried out.

Regarding chitosan ability to avoid bacterial adsorption to hydroxyapatite, the chitosan samples described in the examined papers were not appropriately characterized so it is not possible to conclude which are the properties of the best chitosan or derivative. Authors described their chitosan samples and derivatives as low Mw polymers; but as no clear definition exits for low, medium and high molecular weight chitosan it is not possible to evaluate the polymer size. Moreover, it cannot be dismissed that medium or high molecular weight samples avoid bacterial adsorption since they had not been tested.

Regarding the use of chitosan as sunscreen, it seems that this activity depends on the chitosan sample but from the reported data it is not possible to determine which chitosan property is involved in this activity.

## 5. Conclusions

Chitin, chitosan and its derivatives exhibit many different relevant properties as active ingredient in dental, skin, hair and nails care. Moreover, they have optimal properties to vehiculate active ingredients for the cosmetics and cosmeceutical industry.

These valuable properties are strongly related to the polymer physico-chemical characteristics and therefore an accurate polymer characterization is needed to determine which characteristics are more relevant for a specific application.

It is very remarkable that several properties can be found in a single chitosan derivative, for instance, Carboxymethyl Caprooyl Chitosan (Chitosonic^®^ acid) is a water-soluble derivative with high HLB value; that can form a nano-network structures at higher concentration than 0.5% and can self-assemble into a nanosphere structure at lower concentration than 0.2%. Besides, it has potent antimicrobial activity against gram-positive bacteria, gram-negative bacteria and fungus; moderate DPPH radical scavenging activity and exhibits good hydration activity for absorbing and retaining water molecules [113].

This multi-functional behaviour is quite frequent and implies that each polymer must be characterized not only in terms of its physico-chemical properties but also in terms of functional properties.

An accurate polymer characterization will boost our knowledge in these polymers and promote their use in the industry.

## Figures and Tables

**Table 1:** Statements included in Reed´s cosmeceutical definition.

(i) A Cosmeceutical is a scientifically designed product intended for external application to the human body
(ii) A Cosmeceutical produces a useful and desired result
(iii) A Cosmeceutical has desirable aesthetic properties
(iv) A Cosmeceutical meets rigid chemical, physical and medical standards

**Table 2:** Some potential cosmeceutical ingredients from marine resources and their use [9,10].

Ingredient	Source	Activity/Use
Alginate	Seaweed (brown algae)	Texture and emulsion stabilizer Vehicle for controlled delivery Thickening agent
Fucoidans		Wound-healing
Phlorotannin		Sunscreen and antioxidant activities
Fucoxanthin		UV protective and antioxidant activities
Carrageenan	Seaweed (red algae)	Viscosity altering Thickening agent
MAAs		Antioxidant
Ulvans	Seaweed (green algae)	Antioxidant
Glycogen	Mussel	UVB protection Moisturizing
Aluminium silicate	Sea mud	Absorbent
Squalene	Shark	Skin lubrication
Chitin	Crustaceans shells	Vehicle for controlled delivery Antiaging Skin protecting
Chitosan	Crustaceans shells	Vehicle for controlled delivery Antimicrobial Antioxidant Emulsifying Skin protecting

MAAs: Mycosporine-like amino acids.

**Table 3:** Delivery formulations used in oral care.

Vehicle	Characteristics
Mouth rinses	Simplest vehicle formulation Compatible with most antimicrobial agents
Sprays	Relatively small doses to achieve efficacy Good compliance Easy usage
Dentifrices	Complex formulation Possible interaction among components Tooth brushing with a dentifrice is a well-adopted habit
Gels	Thickened aqueous system Non-abrasive No foaming agents Compatible with relevant antimicrobials Specific devices are needed for applications
Chewing gum/lozenges	Larger contact time Stimulated salivary secretion Useful for patients with low tooth-brushing compliance
Sustained-release formulations/devices	Long-term effect The efficacy is independent of patient compliance

**Table 4:** Hair care products requisites.

Low stickiness
Lack of powdering or flaking
Preferably being clear
Preferably transparent
Preferably glossy
Good film formation
Good holding power
High level of style retention
Prolonged curl retention
Improved combability
Easily removed upon washing the hair

**Table 5:** Effect of chitosan *M*_w_ and deacetylation degree on minimum inhibitory concentrations (MIC) against oral pathogens [27,28,29,30].

Bacterial Strain	Chitosan Properties		MIC mg/mL
	*M*_w_, kDa	DA	
*S. mutants*	1400	0.2	0.08
	1080	0.14	2.5
	624	<0.25	3
	107	0.15–0.25	5
*P. intermedia*	1080	0.14	2.5
	624	<0.25	1
	107	0.15–0.25	3
*P. buccae*	624	<0.25	3
	107	0.15–0.25	1
*T. forythensis*	624	<0.25	1
	107	0.15–0.25	3
*A. actinomycetemcomitans*	1080	0.14	2.5
	624	<0.25	5
	107	0.15–0.25	3
*P. gingivalis*	1080	0.14	0.5
	624	<0.25	1
	272	0.05	3.8
	272	0.16	3.8
	272	0.27	3.6
	107	0.15–0.25	1

MIC: minimum inhibitory concentration. DA: Acetylation degree.

**Table 6:** Use of chitin and chitosan derivatives in oral healthcare [27,34,37,49,50,51,52].

Polymer Derivative	Bacterial Strain	Effect
Ethylenglycol chitin	*S. mutants* *S. sanguis* *S mitis*	Reduce bacterial adsorption on S-HA in vitro Dose dependent effect Better activity on *S. mutants*
Carboxymethyl chitin	*S. mutants* *S. sanguis* *S mitis*	Reduce bacterial adsorption on S-HA in vitro Dose dependent effect Better activity on *S. mutants*
*N*-Carboxymethyl chitosan	*S mutants*	Prevent bacterial adsorption to *HA* in vitro
	*S. sanguis*, *S. gordonii*, *S. constellatus*, *S. anginosus*, *S. intermedius*, *S. oralis*, *S. salivarius*, *S. vestibularis*	Adsorption reduction on HA and S-HA (60%–98%) in vitro
Imidazolyl chitosan	*S mutants*	Prevent bacterial adsorption to HA in vitro
	*S. sanguis*, *S. gordonii*, *S. constellatus*, *S. anginosus*, *S. intermedius*, *S. oralis*, *S. salivarius*, *S. vestibularis*	No effect on bacterial adhesion to HA or S-HA in vitro
Sulphated chitosan	*S. muntants* *S. sanguis* *S. mitis*	Reduce bacterial adsorption on S-*HA* in vitroDose dependent effect. Better activity on *S. mutants*
Phosphorylated chitosan	*S. muntants* *S. sanguis* *S. mitis*	Reduce bacterial adsorption on S-HA in vitro. Dose dependent effect. Better activity on *S. mutants* Plaque reduction Slight plaque buffering capacity
*N*-1hydroxy 3 trimethyl ammonium chitosan HCl	*P. gingivalis* *P. intermedia* *A. actinomycetemcomitans* *S. mutans*	Antibacterial activity in vitro MIC: 0.5–1 mg/mL
Glucosamine Maillard chitosan derivative	*S. mutants* *L. brevis*	CBM *S. mutants* 0.4 mg/mL CBM *L. brevis* 0.5 mg/mL No cytotoxicity in vivo
Water-soluble reduced chitosan	*S. mutans* *S sanguinis*	MIC *S mutans* 1.25 mg/mL MIC *S sanguinis* 10 mg/mL Reduction plaque index Reduction vital fluorescence

MIC: minimum inhibitory concentration. MBC: minimum bactericidal concentration; HA: Hydroxyapatite S-HA Saliva coated hydroxyapatite.

**Table 7:** Polymer requisites in hair care.

Requisite	Chitosan	Water Soluble Derivatives
Heat stability	Up to 170 °C	To be checked
Very good solubility	Only acidic media, depends on DA and Mw	Yes
Compatibility with cosmetic bases	Yes	To be checked
pH stability in the range of 4 to 9	Yes	To be checked
Processability into a variety of products	Yes	Yes
Compatibility with other ingredients and with the packaging materials	Yes	Yes
Free of colour	White to yellowish	To be checked
Neutral or pleasant odour	Yes	Yes
Low volatility	Non-volatile	Non-volatile

**Table 8:** Chitin and chitosan derivatives with hair conditioning and film forming functions according to CosIng Database.

Hair Conditioning	Film Forming
Butoxy CH Carboxybutyl CH Carboxymethyl CH Succinamide CHT Propylsulfonate Hydrolyzed CHT CHT Hydroxypropyltrimonium Chloride CHT Isostearamide Hydroxypropyltrimonium Chloride CHT Lauramide Succinamide CHT Lauroyl Glycinate CHT Rice Branamide Hydroxypropyltrimonium Chloride Sodium Carboxymethyl CHT Lauramide Sodium CHT Caprylamide Hydroxypropylsulfonate Sodium CHT Cocamide Hydroxypropylsulfate Sodium CHT Cocamide Hydroxypropylsulfonate Sodium CHT Isostearamide Hydroxypropylsulfonate Sodium CHT Lauramide Hydroxypropylsulfate Sodium CHT Lauramide Hydroxypropylsulfonate Sodium CHT Rice Branamide Hydroxypropylsulfonate Sodium CHT Stearamide Hydroxypropylsulfonate Carboxymethyl CH	Butoxy CH Calcium CH Carboxybutyl CH Carboxymethyl Caprooyl CHT Carboxymethyl CHT Carboxymethyl CHT Succinamide CHT Adipate CHT Ascorbate CHT Formate CHT Glycolate CHT Lactate CHT PCA Palmitamide Succinamide CHT Propylsulfonate CHT Salicylate CHT Succinamide Hydrolyzed CH Hydroxyethyl CHTT Hydroxypropyl CHT Polyquaternium-29

CHT: Chitosan; CH: Chitin.

**Table 9:** Chitin and Chitosan derivatives with function as a skin conditioning and emollient in CosIng Database.

Skin Conditioning	Emollient
Calcium CHT Carboxybutyl CHT Carboxymethyl Caprooyl CHT Carboxymethyl CHT Succinamide CHT Ascorbate CHT Caprylamide Hydroxypropyl trimonium Chloride Hydrolyzed CHT Hydrolyzed CHT Ferulyl Linoleate Myristoyl/PCA CH Polyquaternium-29 Sodium Carboxymethyl CHT Lauramide Sodium CHT Cocamide Hydroxypropyl sulfate Sodium CH Cocamide Hydroxypropyl sulfonate Sodium CHT Isostearamide Hydroxypropyl sulfonate Sodium CHT Lauramide Hydroxypropyl sulfate Sodium CHT Lauramide Hydroxypropyl sulfonate Sodium CHT Rice Branamide Hydroxypropyl sulfonate Sodium CHT Stearamide Hydroxypropyl sulfonate Sodium Carboxymethyl CH CHT Glycolate CHT Isostearamide Hydroxypropyl trimonium Chloride CHT Lauramide Hydroxypropyltrimonium Chloride CHT PCA Palmitamide Succinamide CHT Propylsulfonate CHT Rice Branamide Hydroxypropyl trimonium Chloride CHT Salicylate Carboxymethyl CH CH glycolate CH sulfate Hydrolyzed CH Mystoil PCA CH	CHT Rice Branamide Hydroxypropyl trimonium Chloride Sodium CHT Caprylamide Hydroxypropyl sulfonate Sodium CHT Cocamide Hydroxypropyl sulfate Sodium CHT Cocamide Hydroxypropyl sulfonate Sodium CHT Isostearamide Hydroxypropyl sulfonate Sodium CHT Rice Branamide Hydroxypropyl sulfonate Sodium CHT Stearamide Hydroxypropyl sulfonate CHT Caprylamide Hydroxypropyl trimonium Chloride

CHT: Chitosan; CH: Chitin.

**Table 10:** Chitin and chitosan derivatives with humectant or moisturizing activity included in CosIng Database.

Humectant	Moisturizing
Carboxymethyl Caprooyl CHT	CHT Rice Branamide Hydroxypropyltrimonium Cl
Carboxymethyl CHT Myristamide	Sodium CHT Cocamide Hydroxypropylsulfate
Carboxymethyl CHT Succinamide	Sodium CH Cocamide Hydroxypropylsulfonate
CHT Hydroxypropyltrimonium Cl	Sodium CHT Isostearamide Hydroxypropylsulfonate
CHT Lauroyl Glycinate	Sodium CHT Rice Branamide Hydroxypropylsulfonate
CHT PCA Palmitamide Succinamide	Sodium CHT Stearamide Hydroxypropylsulfonate
CH sulfate	CHT Caprylamide Hydroxypropyltrimonium Cl
Sodium Carboxymethyl CH	Carboxybutyl CHT
	Carboxymethyl CHT Succinamide
	CHT Propylsulfonate
	Hydrolyzed CHT
	CHT Isostearamide Hydroxypropyltrimonium Cl
	Sodium Carboxymethyl CHT Lauramide
	Sodium CHT Lauramide Hydroxypropylsulfate
	Sodium CHT Lauramide Hydroxypropylsulfonate
	Calcium CHT
	Carboxymethyl Caprooyl CHT
	CHT Ascorbate
	CHT Glycolate
	CHT PCA Palmitamide Succinamide
	CHT Salicylate
	Polyquaternium-29
	CHT Lauramide Hydroxypropyltrimonium Cl
	CHT PCA Palmitamide Succinamide
	Hydrolyzed CHT Ferulyl Linoleate
	Myristoyl/PCA CH

CHT: Chitosan; CH: Chitin.

**Table 11:** Chitin and chitosan derivatives with surfactant, emulsifier and viscosity controlling function.

Surfactant	Emulsifier	Viscosifier
Carboxymethyl Caprooyl CHT CHT Argininamide CHT Lauramide Hydroxypropyl trimonium Cl CHT Stearamide Hydroxypropyl trimonium Cl Sodium Carboxymethyl CHT Lauramide Sodium CHT Caprylamide Hydroxypropyl sulfonate Sodium CHT Cocamide Hydroxypropyl sulphate Sodium CHT Cocamide Hydroxypropyl sulfonate Sodium CHT Lauramide Hydroxypropyl sulphate Sodium CHT Rice Branamide Hydroxypropyl sulfonate Sodium CHT Stearamide Hydroxypropyl sulfonate	CHT Isostearamide Hydroxypropyl trimonium Cl CHT Lauramide Hydroxypropyl trimonium Cl CHT PCA Palmitamide Succinamide CHT Rice Branamide Hydroxypropyl trimonium Cl CHT Stearamide Hydroxypropyl trimonium Cl Sodium Carboxymethyl CHT Lauramide Sodium CHT Caprylamide Hydroxypropyl sulfonate Sodium CHT Cocamide Hydroxypropyl sulphate Sodium CHT Cocamide Hydroxypropyl sulfonate Sodium CHT Lauramide Hydroxypropyl sulphate Sodium CHT Lauramide Hydroxypropyl sulfonate Sodium CHT Rice Branamide Hydroxypropyl sulfonate	Carboxymethyl CH Butoxy CHT Carboxybutyl CHT Carboxymethyl CHT Hydroxyethyl CHT

CHT: Chitosan; CH: Chitin.

**Table 12:** Chitosan derivatives with antioxidant and antimicrobial activity included in CosIng data base.

Antioxidant	Antimicrobial
CHT Ascorbate CHT Glycolate CHT Salicylate Hydrolysed CHT Ferulyl Linoleate	Hydrolysed CHT Ferulyl Linoleate CHT Benzamide

CHT: Chitosan.

**Table 13:** Some general recommendations on chitin, chitosan and derivatives in cosmetics and cosmeceutical applications**.**

Field	Property	Effect of Physico-Chemical Properties
Oral healthcare	ability to inhibit pH fall in dental plaque	In vitro: No effect of *M*_w_ or DA in buffering capacity. In vivo: best sample 3000 Da, DA: 0.05
Oral healthcare	Antimicrobial Activity	Depends on *M*_w_, DA and bacterial strain. Very active against *S. mutants* and other oral *streptococci.*
Oral healthcare	Bacterial adsorption inhibition	Dual-species inhibition (High *M*_w_ :624 kDa, DA < 0.25). Inhibition *S. sobrinus* High Mw, optimum DA 0.40–0.50
Product conservation	Antioxidant	Best activity COS. Chitosanase- COS are preferred over lysozyme-COS
Product conservation	Antimicrobial Activity	Depends on *M*_w_, DA, bacterial strain
Skin care	Antioxidant	Best activity COS Chitosanase-COS are preferred over lysozyme-COS
Skin care (Acne treatment)	Antimicrobial activity	High *M*_w_
Skin/hair care	Humectant	Low DA
Skin/hair care	Moisturizing agent	High *M*_w_ Carboxylmethyl derivatives
Skin care	Sunscreen	Possible effect of the chitosan source

Chitosanase-COS: COS produced by chitosanase; Lysozyme-COS: COS produced by lysozyme, DA: Acetylation degree.
